# Senescence-inducible cell wall and intracellular purple acid phosphatases: implications for phosphorus remobilization in *Hakea prostrata* (Proteaceae) and *Arabidopsis thaliana* (Brassicaceae)

**DOI:** 10.1093/jxb/eru348

**Published:** 2014-08-28

**Authors:** Michael W. Shane, Kyla Stigter, Eric T. Fedosejevs, William C. Plaxton

**Affiliations:** ^1^School of Plant Biology (M084), Faculty of Science, The University of Western Australia, Crawley (Perth) 6009, Australia; ^2^Department of Biology, Queen’s University, Kingston, Ontario, Canada K7L 3N6; ^3^Department of Biomedical and Molecular Sciences, Queen’s University, Kingston, Ontario, Canada K7L 3N6

**Keywords:** *Arabidopsis thaliana*, cell wall hydrolases, *Hakea prostrata*, phosphorus remobilization, Proteaceae, purple acid phosphatase, ribonuclease, senescence.

## Abstract

Despite its agronomic importance, the metabolic networks mediating phosphorus (P) remobilization during plant senescence are poorly understood. Highly efficient P remobilization (~85%) from senescing leaves and proteoid roots of harsh hakea (*Hakea prostrata*), a native ‘extremophile’ plant of south-western Australia, was linked with striking up-regulation of cell wall-localized and intracellular acid phosphatase (APase) and RNase activities. Non-denaturing PAGE followed by in-gel APase activity staining revealed senescence-inducible 120kDa and 60kDa intracellular APase isoforms, whereas only the 120kDa isoform was detected in corresponding cell wall fractions. Kinetic and immunological properties of the 120kDa and 60kDa APases partially purified from senescing leaves indicated that they are purple acid phosphatases (PAPs). Results obtained with cell wall-targeted hydrolases of harsh hakea were corroborated using *Arabidopsis thaliana* in which an ~200% increase in cell wall APase activity during leaf senescence was paralleled by accumulation of immunoreactive 55kDa AtPAP26 polypeptides. Senescing leaves of an *atpap26* T-DNA insertion mutant displayed a >90% decrease in cell wall APase activity. Previous research established that senescing leaves of *atpap26* plants exhibited a similar reduction in intracellular (vacuolar) APase activity, while displaying markedly impaired P remobilization efficiency and delayed senescence. It is hypothesized that up-regulation and dual targeting of PAPs and RNases to the cell wall and vacuolar compartments make a crucial contribution to highly efficient P remobilization that dominates the P metabolism of senescing tissues of harsh hakea and *Arabidopsis*. To the best of the authors’ knowledge, the apparent contribution of cell wall-targeted hydrolases to remobilizing key macronutrients such as P during senescence has not been previously suggested.

## Summary

Targeting of senescence-inducible acid phosphatases and RNases to the cell wall and vacuolar compartments appears to make a crucial contribution to efficient P remobilization networks of senescing tissues of *Hakea prostrata* and *Arabidopsis*.

## Introduction

Senescence is a tightly controlled developmental process involving the induction of senescence-associated genes that play key roles in protein, lipid, and nucleic acid breakdown and nutrient recycling (Thomas, 2013). Studies of the critical process of senescence-induced macromolecule catabolism together with remobilization of released nutrients to developing seeds and growing tissues have focused primarily on nitrogen rather than phosphorus (P) (Buchanan-Wollaston *et al.*, 2003; Lim *et al.*, 2007; Thomas, 2013). P-deficient plants remobilize P from their older leaves and roots to younger tissues, but the metabolic networks mediating P remobilization during senescence are poorly understood (Buchanan-Wollaston *et al.*, 2003; Robinson *et al.*, 2012*a*). However, maximizing the effectiveness of P remobilization from senescing tissues will probably make an important contribution to development of crops with enhanced P use efficiency (PUE) since most modern crop varieties exhibit relatively poor P remobilization efficiency during leaf senescence (Veneklaas *et al.*, 2012). Even a small improvement in this capacity would probably lead to an important reduction in the use of non-renewable P-containing fertilizers in agriculture, thus reducing P_i_ run-off and pollution of aquatic ecosystems (Veneklaas *et al.*, 2012).

Cellular P occurs in various pools, including nucleic acids, phospholipids, phosphorylated metabolites, and P_i_. Ribosomal RNA (rRNA) is the largest organic-P (P_o_) component of mature leaves, accounting for up to 60% of their total P_o_ content (Raven, 2012; Veneklaas *et al.*, 2012). The DNA content remains relatively constant as leaves senesce, whereas RNA levels steadily decrease owing to the up-regulation of specific RNase isozymes (Lers *et al.*, 1998; Buchanan-Wollaston *et al.*, 2003). Genomic, proteomic, and biochemical approaches using the model plant *Arabidopsis thaliana* have made an important contribution to the identification and characterization of genes involved in plant senescence and PUE. For example, the S-like RNase AtRNS2 and the purple acid phosphatase (PAP) AtPAP26 are both induced during *Arabidopsis* senescence or P deprivation (Bariola *et al.*, 1999; Robinson *et al*., 2012*a*, *b*). AtRNS2 has been localized to the cell wall, endoplasmic reticulum, and cell vacuole, where it mediates degradation of rRNA (Bariola *et al.*, 1999; Borderies *et al.*, 2003; Hillwig *et al.*, 2011).

Acid phosphatases (APases, EC 3.1.3.2) catalyse the release of P_i_ from a broad and overlapping spectrum of phosphomonoesters with an acidic pH optimum. PAPs are an important class of plant APases that function in P_i_ production and recycling (Tran *et al.*, 2010*a*). AtPAP26, one of 29 PAP isozymes encoded by the *Arabidopsis* genome, is targeted to the cell vacuole where it has a crucial P-scavenging function during leaf senescence or nutritional P deprivation (Hurley *et al.*, 2010; Robinson *et al*., 2012*a*, *b*; Wang *et al.*, 2014). Kinetic studies with purified AtPAP26 demonstrated that this PAP is well suited for P scavenging as it effectively cleaves P_i_ from a broad range of substrates with a high catalytic efficiency (Veljanovski *et al.*, 2006; Tran *et al.*, 2010*b*). During P deficiency, AtPAP26 is also up-regulated and secreted into the cell wall, where it was hypothesized to scavenge apoplastic P_i_ by hydrolysing P-esters leaked from the cytoplasm (Robinson *et al.*, 2012*b*). However, the possible involvement of cell wall-localized acid hydrolases such as AtPAP26 or AtRNS2 in nutrient remobilization during senescence is currently unknown. The cell wall is a dynamic structure intimately involved in plant growth and development, abiotic stress response, and interactions with pathogens and symbionts. The complexity and importance of the cell wall are reflected by the large number of genes suspected to play a role in its biogenesis, assembly, and modifications (Albenne *et al.*, 2013). Although cell wall proteins represent only ~10% of the cell wall mass, they comprise hundreds of different proteins that function as cell wall-modifying enzymes, structural proteins, or defence proteins synthesized in response to biotic or abiotic stress (Albenne *et al.*, 2013).

The proteoid root-forming *Hakea prostrata* R.Br. (harsh hakea) is one of many non-mycorrhizal Proteaceae species well adapted to the severely nutrient-impoverished soils of south-western Australia (Lamont, 2003; Shane *et al*., 2003, 2013; Lambers *et al.*, 2014). The metabolic mechanisms that mediate extremely efficient P remobilization that characterizes senescing harsh hakea tissues (≥85%; Shane *et al.*, 2004) are unknown. The objectives of the current study were therefore to: (i) investigate the impact of senescence on APase and RNase activities in the cell wall and corresponding intracellular fractions of leaves and proteoid roots of harsh hakea; and (ii) employ wild-type *Arabidopsis* and an *atpap26* loss-of-function mutant to assess the possible involvement of cell wall-targeted AtPAP26 isoforms during senescence-induced P recycling. Parallel up-regulation and dual targeting of PAPs and RNases to the cell wall and intracellular compartments was demonstrated in senescing tissues of both species. This is hypothesized to contribute to efficient P remobilization during senescence by effectively recycling P_i_ from endogenous P_o_ compounds.

## Materials and methods

### Plant material and growth conditions

**Fig. 1.**
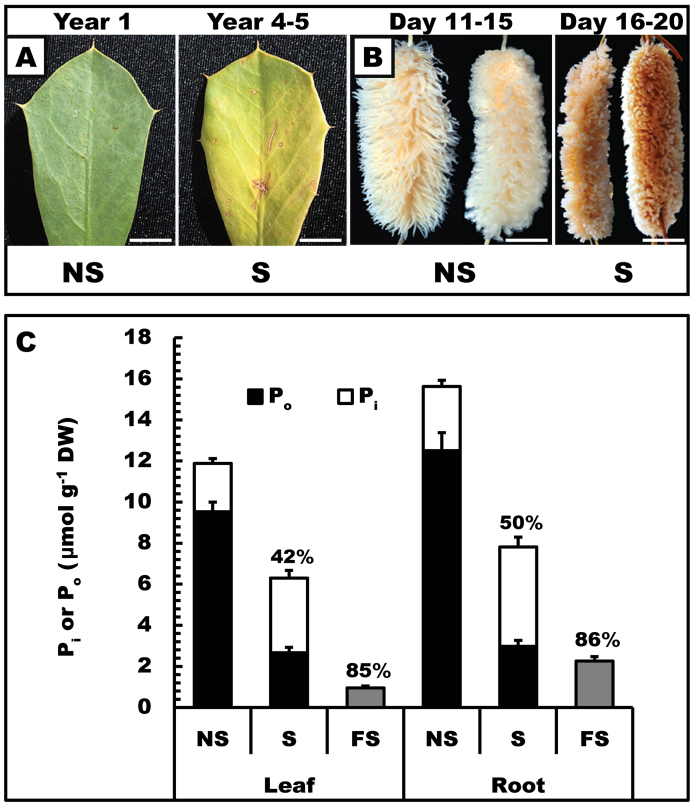
Leaves and proteoid roots of harsh hakea have very different lifespans but similar P remobilization efficiencies. Non-senescing (NS), senescing (S), and fully senesced (FS) leaves and proteoid roots were harvested as described in the Material and methods. (A) Senescing leaves were 4–5 years old and 50–75% yellow compared with 1–2 year old non-senescing, mature green leaves. Scale bar=0.75cm. (B) Senescing proteoid roots were 18–22 d old and greyish-brown compared with mature 11–15-day-old non-senescing mature, bright white, proteoid roots. Scale bar=1cm. (C) P_i_ and P_o_ were determined for non-senescing and senescing tissues, whereas total P was determined for fully senesced tissues. Values above the bars indicate the percentage of P that was remobilized. The asterisks denote statistically significant differences (*P*<0.05). All values represent the mean (±SE) of triplicate determinations of *n*=4 biological replicates.

Non-senescent, senescing, and fully senesced leaves were harvested from mature harsh hakea (*Hakea prostrata* R.Br.) plants growing in a natural bushland at Shenton Park Field Station (31°S57’00.27’’, 115°E47’52.14’’, University of Western Australia). Proteoid roots (Fig. 1B) were harvested from hydroponically cultivated harsh hakea plants propagated in a glasshouse at the University of Western Australia as described in Shane *et al.* (2013). The non-senescing and senescing proteoid roots correspond to stages III and IV (Shane *et al.*, 2013), while fully senesced proteoid roots were ≥25 d old with no detectible respiration. Harvested tissues were immediately frozen in liquid N_2_. Samples were transported on dry ice to Queen’s University (Kingston, Canada) and stored at –80 °C.

Wild-type and homozygous *atpap26* T-DNA insertion mutant seeds (Hurley *et al.*, 2010) of *Arabidopsis thaliana* (Col-0 ecotype) were sown in a standard soil mixture (Sunshine Aggregate Plus Mix 1; SunGro, Vancouver, BC, Canada) and stratified for 3 d at 4 °C before being moved to growth chambers (Model MTR30; Conviron, Winnipeg, MB, Canada), where plants were cultivated at 23 °C (16:8 photoperiod at 100 μmol m^–2^ s^–1^ photosynthetically active radiation). Plants were fertilized biweekly by subirrigation with 0.25× Hoagland’s solution. Senescence in mature fully expanded rosette leaves of 28-day-old plants was induced according to Robinson *et al.* (2012*a*). Fully expanded leaves (up to two per plant) of synchronously growing plants were covered with aluminium foil. After 6–9 d, leaves were determined to be senescing when they appeared 90–100% yellow. Brown/shrivelled leaves were classified as being fully senesced. Harvested leaves were immediately frozen in liquid N_2_ and stored at –80 °C.

### Preparation of intracellular and cell wall extracts optimized for harsh hakea

Quick-frozen tissues were ground to a powder under liquid N_2_ using a mortar and pestle, and homogenized [leaves and roots=1:4 and 1:1.5 (w/v), respectively] using a PT-3100 Polytron with ice-cold buffer A containing 50mM imidazole-HCl (pH 6.0), 0.1% (v/v) Triton X-100, 10% (v/v) glycerol, 10mM thiourea, 2mM MgCl_2_, 2% (w/v) polyethylene glycol 8000, 1% (w/v) polyvinyl(polypyrrolidone) (PVPP), 1% (w/v) polyvinylpyrrolidone (PVP), and 1mM phenylmethylsulphonyl fluoride. Homogenates were centrifuged at 17 500 *g* for 15min at 4 °C. Supernatants were designated as the intracellular fraction and desalted through Zeba™ spin desalting columns (Fisher Scientific, Toronto, ON, Canada) equilibrated in extraction buffer lacking PVPP and PVP. The pellets were washed twice via resuspending them (1:10; w/v) in 50mM TRIS-HCl (pH 7.4) containing 10mM MgCl_2_ and 1% (v/v) Triton X-100, and centrifugation as above. Pellets were washed an additional four times in the same buffer lacking Triton X-100. Cell wall extracts were extracted from insoluble pellets with 1M NaCl in 40mM TRIS-HCl (pH 7.4) containing 10mM MgCl_2_, centrifuged as above, concentrated using Macrosep^®^ Advance (Mississauga, ON, Canada) centrifugal device units (10kDa cut-off) to a protein concentration of ~2mg ml^–1^, filtered through 0.45 μm syringe filters, and dialysed overnight against 500ml of 40mM TRIS-HCl (pH 7.4) containing 10mM MgCl_2_.

### Preparation of cell wall extracts optimized for *Arabidopsis*

Leaves were powdered under liquid N_2_ and homogenized (1:15, w/v) using a mortar and pestle in ice-cold homogenizing buffer [25mM HEPES-KOH (pH 7.4) containing 10mM MgCl_2_, 1mM EDTA, 1mM dithiothreitol, 1% (v/v) Triton X-100, and 1% (w/v) PVPP]. Extracts were clarified by centrifugation for 20min at 14 000 *g* and 4 °C, and supernatants were collected as the intracellular extracts. Pellets were washed three times by resuspending in homogenizing buffer and centrifuging as above. Extraction of cell wall proteins from the insoluble fractions was performed using a Polytron and 0.2M CaCl_2_ in 5mM acetate-NaOH (pH 4.6), followed by incubation at 24 °C for 15min. The mixtures were centrifuged as above, and supernatants were collected as the cell wall extract (Robinson *et al.*, 2012*b*). Pellets were re-extracted twice with the same buffer and extracts were pooled. Intracellular and cell wall extracts were filtered through Miracloth before concentration as above to at least 2mg ml^–1^. Cell wall extracts were dialysed overnight against 500ml of 40mM HEPES-KOH (pH 7.4) containing 10mM MgCl_2_, 1mM EDTA, 1mM dithiothreitol, and 1% (v/v) Triton X-100.

### Enzyme and protein assays

All enzyme assays were linear with respect to time and concentration of enzyme assayed. APase activity was determined by coupling the hydrolysis of phospho*enol*pyruvate (PEP) to pyruvate to the lactate dehydrogenase reaction at 25 °C and continuously monitoring NADH oxidation at 340nm using a Molecular Devices Spectramax Plus microplate spectrophotometer (Sunnyvale, CA, USA). The optimized assay mix contained 50mM sodium acetate (pH 5.0), 2.5mM PEP, 5mM MgCl_2_, 0.2mM NADH, and 3U ml^–1^ of desalted rabbit muscle lactate dehydrogenase in a final volume of 0.2ml. Assays were corrected for any background NADH oxidation by omitting PEP from the reaction mixture. Substrate selectivity studies were performed by quantifying the P_i_ released by the APase reaction (Tran *et al.*, 2010*b*). Controls were run for background amounts of P_i_ present at each substrate concentration tested. To calculate activities, a standard curve over the range 1–133 nmol P_i_ was constructed for each set of assays. One unit of APase activity was defined as the amount of enzyme resulting in the hydrolysis of 1 μmol of substrate min^–1^ at 25 °C (Veljanovski *et al.*, 2006). RNase activity was determined using a modification of the procedure of Fennoy *et al.* (1997) and the following assay mixture: 50mM imidazole-HCl (pH 7.0) containing 0.4% (w/v) Torula yeast RNA (Sigma-Aldrich, Canada) in a final volume of 1ml, and transferred to a water bath at 35 °C. Aliquots (200 μl) were removed every 15min for 1h and immediately added to 40 μl of a stop solution containing 25% (v/v) perchloric acid and 0.75% (w/v) uranyl acetate. Samples were incubated on ice for 5min and centrifuged at 17 500 *g* for 5min at 4 °C. Supernatants were diluted 40-fold with water and the *A*_260_ determined. One unit of RNase activity was defined as the amount of enzyme resulting in an increased A260 of 1.0 absorbance units owing to the release of acid-soluble nucleotides. Protein concentrations were determined using a Coomassie Blue G-250 dye binding method (Veljanovski *et al.*, 2006) with bovine γ-globulin as the standard.

### Buffers used during APase purification

All buffers were adjusted to their respective pH at 25 °C. Buffer B contained 50mM sodium acetate-KOH (pH 5.8) and 1mM dithiothreitol. Buffer C contained 50mM sodium acetate-KOH (pH 5.8), 1mM dithiothreitol, and 10% (v/v/) ethylene glycol. Buffer D contained 50mM sodium acetate-KOH (pH 5.8), 1mM dithiothreitol, and 1mM EDTA. Buffer E contained 20mM TRIS-HCl (pH 8.0) and 1mM dithiothreitol.

### Partial APase purification from senescing harsh hakea leaves

Both chromatography steps were carried out at room temperature (24 °C) using an ÄKTA FPLC system (GE Healthcare Bio-Sciences Inc., Baie-D’Urfé, QC, Canada). Quick-frozen senescing leaves (40g) were ground under liquid N_2_ using a mortar and pestle, homogenized in ice-cold buffer A (1:4; w/v) using a Polytron, and centrifuged at 4 °C and 18 000 *g* for 20min. The supernatant was brought to 25% (w/v) polyethylene glycol 8000, stirred for 1h at 4 °C, and centrifuged. The resulting pellet was resuspended in 15ml of buffer B, brought to 35% (saturation) (NH_4_)_2_SO_4_, stirred for 30min at 4 °C, and centrifuged. The pellet was resuspended again in buffer B, brought to 35% (saturation) (NH_4_)_2_SO_4_, and loaded at 2ml min^–1^ on to a column (1×1.0cm) of butyl-Sepharose 4 Fast Flow (GE Healthcare Bio-Sciences Inc.) pre-equilibrated in buffer B. The column was washed until the *A*_280_ decreased to baseline, and APase activity was eluted with a linear gradient (50ml) of a simultaneously decreasing concentration of buffer B (100–0%) and increasing concentration of buffer C (0–100%). Pooled peak activity fractions were exchanged into buffer D and concentrated to 3ml using AMICON Ultra-15 ultrafiltration devices (10kDa cut-off), and applied at 0.5ml min^–1^ onto an HR 5/5 Mono-S column (GE Healthcare Bio-Sciences Inc.) pre-equilibrated with buffer D. The column was washed until the *A*_280_ decreased to baseline and APase (designated as HpPAP1) was eluted with a linear gradient (50ml) of 0–0.5M KCl in buffer D. Peak activity fractions were pooled and concentrated as above to 0.4ml. APase activity in the unbound Mono-S flow-through fractions was exchanged to buffer E, concentrated as above to 1.5ml, and applied at 0.5ml min^–1^ onto a Mono-Q GL 5/50 column (GE Healthcare Bio-Sciences Inc.) pre-equilibrated with buffer E. The column was washed until the *A*_280_ decreased to baseline and APase (designated as HpPAP2) was eluted with a linear gradient (50ml) of 0–1M KCl in buffer E. Peak activity fractions were pooled and concentrated as above to 0.4ml. Aliquots (25 μl) of partially purified HpPAP1 and HpPAP2 were frozen in liquid N_2_, and stored at –80 °C.

### Protein electrophoresis, immunoblotting, and in-gel acid phosphatase activity staining

Non-denaturing PAGE and SDS–PAGE, as well as immunoblotting and chromogenic detection of antigenic polypeptides using an alkaline phosphatase-tagged secondary antibody, were conducted as previously described (Tran *et al.*, 2010*b*). APase activity was visualized following non-denaturing PAGE by in-gel activity staining. Following electrophoresis, the gel was incubated for 20min at 25 °C in 40mM TRIs-HCl (pH 9.0), 2mM EDTA, and 1% (w/v) casein, and then for 20min in 100mM Na-acetate (pH 5.3). APase activity staining bands were revealed by placing gels in 100mM Na-acetate (pH 5.3) containing 10mM MgCl_2_, 0.02% (w/v) Fast Garnet GBC salt, and 0.02% (w/v) β-naphthyl-P. All PAGE and immunoblot results were replicated a minimum of three times, with representative results shown in the various figures.

### Leaf free P_i_ and total P

Free P_i_ in leaves was extracted and quantified as previously described (Delhaize and Randall, 1995). For analysis of total P, leaves were digested in concentrated HNO_3_:HClO_4_ (3:1) at 175 °C and the P concentration was determined using the Malachite green method (Motomizu *et al.*, 1983). Organic phosphorus (P_o_) was estimated by subtracting P_i_ from total P. Leaf P resorption efficiency was calculated according to % efficiency=[(total [P] mature leaf–total [P] senesced leaf)/total [P] mature leaf]×100).

### Statistics

All values are presented as means ±SE. Data were analysed using the Student’s *t*-test, and deemed significant if *P*<0.05.

## Results and Discussion

### Influence of senescence on P remobilization in harsh hakea leaves and proteoid roots

The lifespan of harsh hakea leaves was estimated to be 4–5 years, in contrast to 20–25 d for the proteoid roots (Fig. 1A, B). These results agree with previous studies for a range of Proteaceae from south-western Australia (Witkowski *et al.*, 2001; Denton *et al.*, 2007; Shane *et al.*, 2013), and also highlight a marked contrast in the time interval from senescence initiation to death between leaves (2–3 months) and proteoid roots (5–7 d) (Fig. 1A, B). In a Mediterranean climate and severely P-impoverished environment such as south-western Australia, many native plants have long-lived leaves, which improves their overall PUE. For their proteoid roots, however, living longer than several weeks would elicit a carbon drain without P gains (Shane and Lambers, 2005). As expected, cells with thick walls were prominent in cross-sections of tough leaves of harsh hakea compared with proteoid rootlets (Supplementary Fig. S1A, B available at *JXB* online). Dry matter content of leaves [0.45g dry weight (DW) g^–1^ fresh weight (FW)] was ~10-fold greater than that of proteoid roots (0.05g DW g^–1^ FW) (Supplementary Fig. S1C at *JXB* online), and is consistent with earlier studies (Shane and Lambers, 2005; Sulpice *et al.*, 2014).

To assess the influence of senescence on P remobilization from harsh hakea leaves and proteoid roots, total P was fractionated into P_i_ and P_o_. The P remobilization efficiency of leaves and proteoid roots was ≥85% (Fig. 1C), corroborating previous studies (Shane *et al.*, 2004; de Campos *et al*., 2013). For non-senescing tissues, P_o_ comprised the major fraction (~80%) of total [P] (Fig. 1C). Interestingly, for the senescing tissues, the fraction of [P_o_]/[total P] was ~0.4 (Fig. 1C), demonstrating that senescing leaves and proteoid roots had been collected at the approximate mid-point of P remobilization (Fig. 1C) when the breakdown of P_o_ substrates exceeded translocation of P_i_ out of the senescing tissues. At this stage of senescence, ~75% of the original P_o_ had disappeared and free P_i_ had doubled (Fig. 1C). The findings suggest that cell wall thickness, tissue longevity, and rate of senescence exert little influence on P remobilization efficiency between the functionally distinct, short-lived proteoid roots and relatively long-lived (harsh hakea) or short-lived *Arabidopsis* foliage (Fig. 1C).

### Influence of senescence on intracellular and cell wall-targeted acid phosphatases and RNases of harsh hakea

**Fig. 2.**
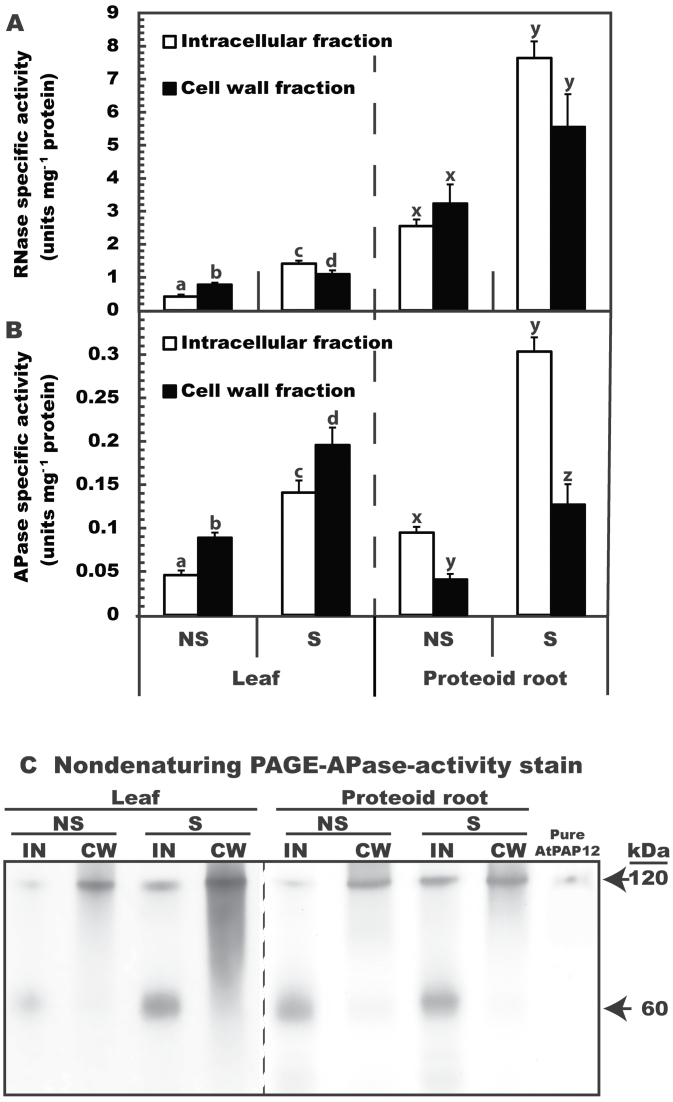
Influence of leaf and proteoid root senescence on RNase and APase activities of harsh hakea. (A, B) All values represent the mean (±SE) RNase (A) or APase (B) specific activity of triplicate determinations of clarified extracts from *n*=5 biological replicates. Different letters above the bars denote statistically significant differences between non-senescing (NS) and senescing (S) intracellular (IN) and cell wall (CW) fractions for leaves and proteoid roots (*P*<0.05). (C) Intracellular and cell wall protein extracts (8 μg per lane) of leaves and proteoid roots as well as purified AtPAP12 (0.5 μg) from P-deprived *Arabidopsis* suspension cells (Tran *et al.*, 2010*b*) were resolved by non-denaturing PAGE and subjected to in-gel APase activity staining.

The cell wall extracts were free of contaminating intracellular proteins as indicated by the absence of cytoplasmic marker proteins (aldolase and PEP carboxylase) on immunoblots of cell wall fractions (Supplementary Fig. S2A at *JXB* online). Comparison of the intracellular and cell wall fractions on protein-stained SDS gels indicated clear differences in their respective proteomes (Supplementary Fig. S2B). The 200–300% up-regulation of RNase and APase specific activities in intracellular and cell wall fractions of senescing leaves and proteoid roots (Fig. 2A, B) was paralleled by significant P remobilization during senescence (Fig. 1C). The specific activities of intracellular and cell wall RNase in harsh hakea were at least 400% greater in proteoid roots than in leaves (Fig. 2A). It is unknown whether this was due to the potential additional role of proteoid roots in extracellular P_i_ scavenging from the soil’s RNA pool and/or to higher relative amounts of RNA in proteoid root cells. Relatively low RNA levels reported for mature non-senescing harsh hakea leaves are considered an adaptive mechanism that increases photosynthetic PUE (Sulpice *et al.*, 2014). RNA levels in proteoid roots have not been determined, but might be expected to be higher, given their much more rapid rate of development and turnover, greater protein concentration, and potentially greater metabolic activity (Shane *et al*., 2004, 2013). In contrast, cell wall APase activity in either non-senescing or senescing leaves was significantly greater than that of proteoid roots (Fig. 2B). It is possible that the difference might be made up by APases excreted in to the rhizosphere by the proteoid roots, and additional studies will be required to assess the cell wall proteomes of leaves versus proteoid roots.

The present results suggest a key P remobilization role for intracellular and cell wall-targeted hydrolases during leaf and proteoid root senescence in harsh hakea. This is hypothesized to contribute to the overall high PUE of harsh hakea, and may apply to similar taxa well adapted to P-impoverished natural habitats (Lambers *et al*., 2011; 2014; Shane *et al.*, 2013). This senescence-induced alteration in P metabolism is potentially very important for proteoid roots which represent significant P sinks but have relatively short lifespans (Fig. 1) (Shane *et al.*, 2004).

### Senescence-inducible acid phosphatases of harsh hakea are purple acid phosphatases

Enhanced APase activity during senescence of harsh hakea leaves and proteoid roots was correlated with the up-regulation of a pair of APase activity-staining bands following non-denaturing PAGE of clarified extracts; a 120kDa band was abundant in both intracellular and cell wall proteomes, whereas a 60kDa band was only detected in the intracellular proteomes (Fig. 2C). Absence of the 60kDa activity-staining band in the cell wall extracts provides further evidence that they were uncontaminated by intracellular proteins. Although extracts from senescing tissues exhibited greater APase activity staining, the major activity-staining bands were similar in extracts from senescing and non-senescing tissue (Fig. 2C). The higher molecular weight APase activity-staining bands co-migrated with AtPAP12 purified from the secretome (cell culture filtrates) of P-deprived *Arabidopsis* suspension cells. Native AtPAP12 exists as a 120kDa homodimer composed of identical 60kDa subunits covalently linked by a disulphide bond (Tran *et al.*, 2010*b*).

**Fig. 3.**
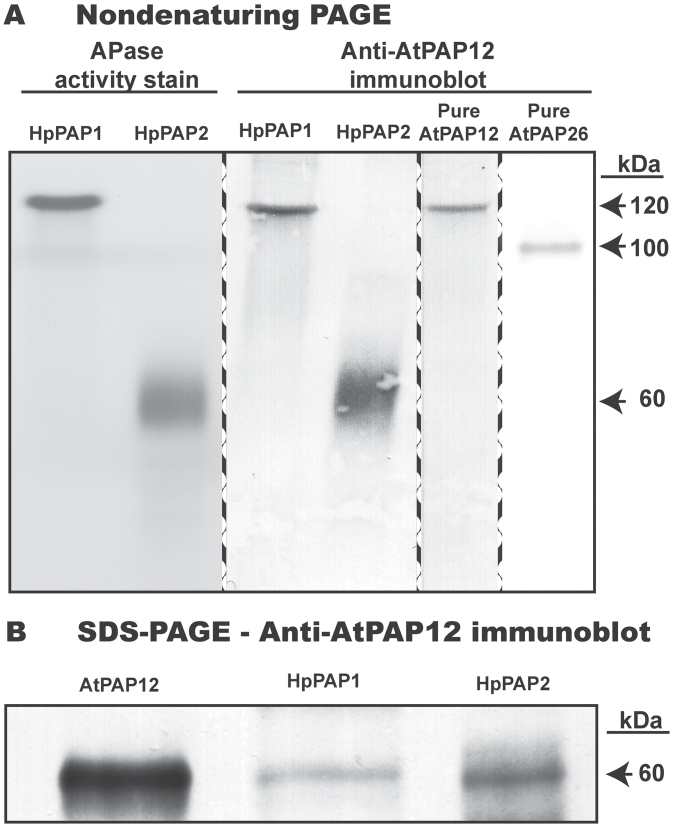
Non-denaturing PAGE and immunoblot analysis of intracellular APases (HpPAP1 and HpPAP2) partially purified from senescing leaves of harsh hakea. (A) Final HpPAP1 and HpPAP2 preparations (1 μg per lane), and homogeneous AtPAP12 and AtPAP26 (0.25 μg per lane) purified from P-deprived *Arabidopsis* suspension cells (Veljanovski *et al.*, 2006; Tran *et al.*, 2010*b*) were resolved by non-denaturing PAGE and subjected to in-gel APase activity staining and/or immunoblotting with anti-AtPAP12 immune serum as indicated. (B) HpPAP1 and HpPAP2 (0.5 μg per lane) and purified AtPAP12 (0.1 μg) were subjected to SDS–PAGE and immunoblotting with anti-AtPAP12.

Intracellular APase activity was resolved as two distinct peaks (HpPAP1 and HpPAP2) during fast protein liquid chromatography (FPLC) of a butyl-Sepharose-enriched senescing leaf extract on a Mono-S cation-exchange column. HpPAP2 failed to bind to a Mono-S column, whereas HpPAP1 was bound and eluted following application of a linear KCl gradient. HpPAP1 and HpPAP2 were purified to final specific APases activities of 0.40U mg^–1^ and 3.4U mg^–1^, respectively (Supplementary Table S1 at *JXB* online). These values are far lower than specific activities of 110U mg^–1^ and 1710U mg^–1^ reported for AtPAP12 and AtPAP26 isozymes purified to homogeneity from culture media of P-deprived *Arabidopsis* suspension cells (Tran *et al.*, 2010*b*). SDS–PAGE followed by protein staining confirmed that the final HpPAP1 and HpPAP2 preparations were relatively impure (results not shown). However, non-denaturing PAGE of partially purified HpPAP1 and HpPAP2 yielded single 120kDa and 60kDa APase activity-staining bands, respectively, that cross-reacted with anti-(recombinant AtPAP12) immune serum (Fig. 3A). Anti-AtPAP12 immune serum is monospecific to high molecular weight members of the plant PAP family, but not to individual PAP isozymes (Tran *et al.*, 2010*b*). To determine subunit molecular masses of HpPAP1 and HpPAP2, samples were resolved by SDS–PAGE and subjected to immunoblotting with the anti-AtPAP12 immune serum (Fig. 3B). In each case an ~60kDa immunoreactive polypeptide was observed, similar to the subunit sizes of high molecular weight plant PAPs (Tran *et al*., 2010*a*, *b*). The combined results indicate that native HpPAP1 exists as a 120kDa homodimer composed of 60kDa subunits, whereas native HpPAP2 is a 60kDa monomer (Fig. 3B).

HpPAP1, but not HpPAP2, was activated (~70%) by Mg^2+^ (Supplementary Table S2 at *JXB* online). There was no effect on the activity of either HpPAP when the reaction mixture (lacking Mg^2+^) contained 5mM EDTA. The most potent HpPAP inhibitors were molybdate, vanadate, and fluoride, with some variation in inhibition between the two isoforms (Supplementary Table S1). Similar findings have been reported for other plant PAPs (Bozzo *et al.*, 2002; Veljanovski *et al.*, 2006; Tran *et al*., 2010*a*, *b*). In particular, the APase activity of HpPAP1 and HpPAP2 was insensitive to 10mM l-tartrate, a diagnostic kinetic feature of all PAPs (Tran *et al.*, 2010*a*). The relative insensitivity of HpPAP1 and HpPAP2 to feedback inhibition by P_i_ (Supplementary Table S1) was surprising, given that PAPs from P-deprived *Arabidopsis* (Veljanovski *et al.*, 2006) and tomato (Bozzo *et al.*, 2002) are subject to potent product inhibition by P_i_. However, such feedback inhibition could be counterproductive during senescence when P-ester hydrolysis needs to continue in the presence of elevated P_i_ levels (Fig. 1C).

The overlapping, but non-identical, substrate selectivities (Table 1), and pH activity profiles of HpPAP1 and HpPAP2 (optimal activities occurring in the range of pH 4.4–5.2, Supplementary Fig. S3 at *JXB* online) support the hypothesis that senescence-inducible PAPs of harsh hakea function to scavenge P_i_ efficiently from a wide range of phosphomonoesters over a broad pH range. Although neither PAP showed activity with phytic acid, they both readily hydrolysed glycerol-3-P, ATP, ADP, and P-tyrosine as substrates (Table 1). However, some clear differences in their substrate selectivity were noted; for example, HpPAP2 exhibited much greater activity with phenyl-P and PP_i_ relative to HpPAP1.

**Table 1.** Substrate selectivity of intracellular APases purified from senescing leaves of harsh hakeaAPase activity was determined with 5mM of each compound using the spectrophotometric P_i_ assay described in the Materials and methods. Activity is expressed relative to the rate of P_i_ hydrolysis from 5mM PEP set at 100%. All values represent the means of *n*=4 separate determinations and are reproducible to within 10% of the mean value.

Substrate	Relative activity	
	*HpPAP1*	*HpPAP2*
PEP	100	100
β-Naphthyl-P	117	138
ATP	114	61
P-tyrosine	106	96
ADP	75	60
Phenyl-P	64	102
NaPP_i_	58	117
Glycerol-3-P	40	50
6-P-gluconate	40	11
3-P-glycerate	35	50
P-serine	23	3
Glucose-6-P	20	40
AMP	6	19
Fructose-6-P	4	5
Glycerol-2-P	3	8

### Influence of senescence on cell wall-targeted acid phosphatase (AtPAP26) and RNase of *Arabidopsis*

**Fig. 4.**
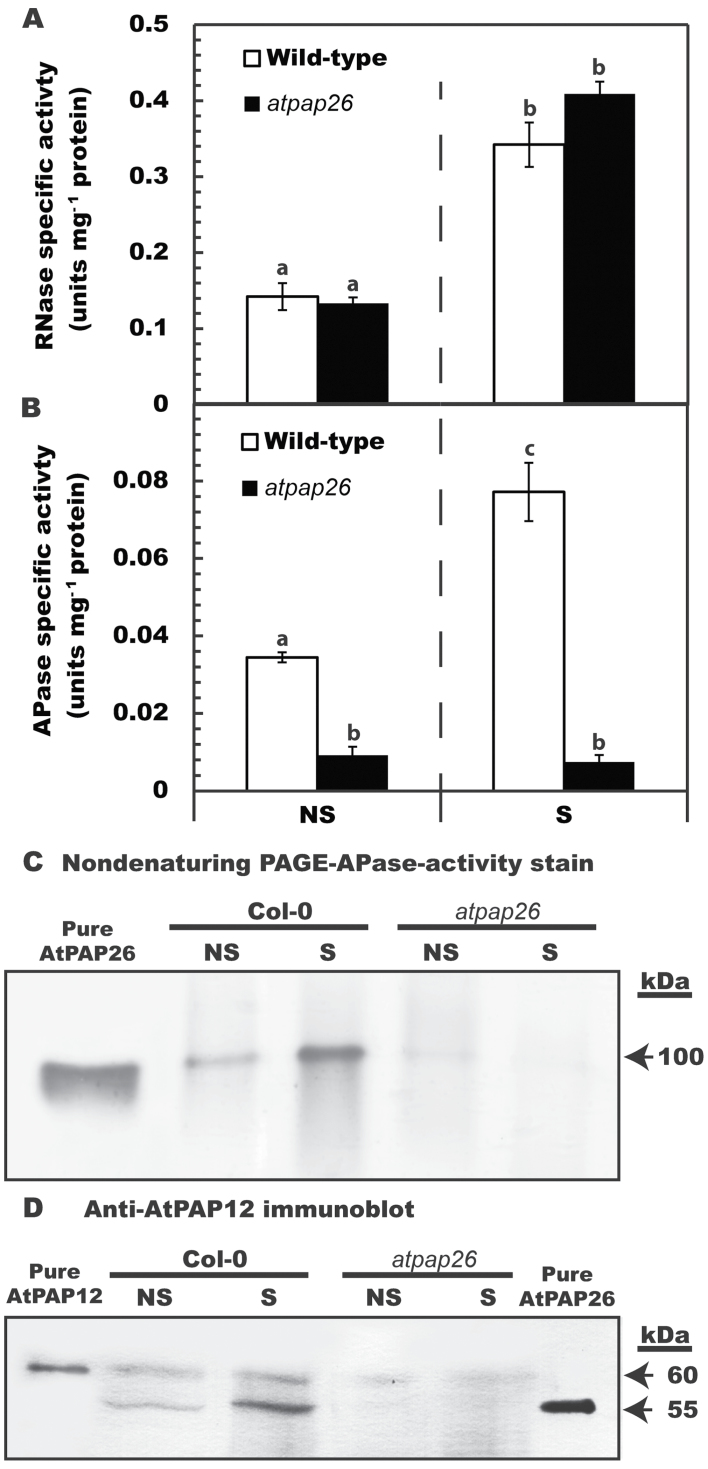
Influence of leaf senescence on RNase and APase activities, and relative levels of AtPAP26 polypeptides in cell wall extracts of wild-type and *atpap26* mutant plants. *Arabidopsis* leaves were considered to be senesced when they appeared as 90–100% yellow which required 6 d and 9 d of dark treatment for wild-type and *atpap26* leaves, respectively (Robinson *et al.*, 2012*a*). (A, B) Concentrated cell wall extracts of non-senescing (NS) or senescing (S) leaves were assayed for RNase (A) or APase (B) activities. All values represent the mean (±SE) of triplicate determinations on *n*=3 biological replicates. The letters denote statistically significant differences between cell wall fractions of NS versus corresponding S samples (*P*<0.05). (C) Non-denaturing PAGE of cell wall extracts (25 μg per lane) and homogeneous AtPAP26 (15ng) was followed by in-gel APase activity staining. (D) SDS–PAGE of cell wall extracts (30 μg per lane) and purified native AtPAP12 and AtPAP26 (15ng each) was followed by immunoblot analysis with anti-AtPAP12 immune serum.

When *Arabidopsis* is subjected to P deficiency, AtPAP26 is up-regulated and dual targeted to the cell vacuole and cell wall to recycle and scavenge P_i_ from intra- and extracellular P_o_ sources, respectively (Veljanovski *et al.*, 2006; Hurely *et al*., 2010; Robinson *et al.*, 2012*a*; Wang *et al.*, 2014). Although AtPAP26 also plays a pivotal P remobilization function during dark-induced leaf senescence of P-sufficient *Arabidopsis*, it was not determined if cell wall-targeted AtPAP26 is also up-regulated during senescence (Robinson *et al.*, 2012*b*). In the current investigation, cell wall RNase activity in senescing leaves of the wild type (Col-0) or an *atpap26* loss-of-function mutant was increased by ~300% relative to non-senescing controls (Fig. 4A). Based upon the findings of Borderies *et al.* (2003), along with pronounced induction of *AtRNS2* in senescing *Arabidopsis* leaves (Robinson *et al.*, 2012*b*), it is hypothesized that the cell wall RNase activity measured in the current study was due to AtRNS2.

Senescing Col-0 leaves also exhibited a significant (~200%) increase in cell wall-localized APase activity (Fig. 4B), whereas cell wall APase activity of the *atpap26* mutant was very low in non-senescing controls and remained unchanged during senescence (Fig. 4B). When cell wall proteins were resolved by non-denaturing PAGE and subjected to in-gel APase activity staining, a 100kDa band was observed that showed a pronounced increase in senescing Col-0 leaves, and that co-migrated with the 100kDa native AtPAP26 homodimer previously purified from P-deprived *Arabidopsis* suspension cells (Fig. 4C). The 100kDa APase (AtPAP26) activity staining band was absent in cell wall extracts from *atpap26* leaves (Fig. 4C). Immunoblotting confirmed the accumulation of immunoreactive 55kDa AtPAP26 polypeptides in cell wall extracts of senescing Col-0 leaves, whereas AtPAP26 polypeptides were absent on immunoblots of *atpap26* cell wall extracts (Fig. 4D). The *Arabidopsis* cell wall extracts used in the previous and present studies were free of contamination by a cytoplasmic marker protein (PEP carboxylase), and protein-stained SDS gels indicated clear differences between the leaf cell wall and corresponding intracellular proteomes (Supplementary Fig. S1D at *JXB* online) (Robinson *et al.*, 2012*a*).

### Concluding remarks

**Fig. 5.**
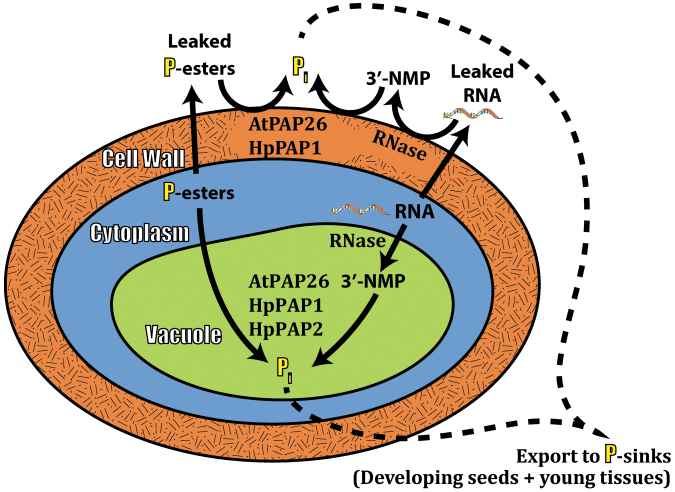
Model summarizing senescence-induced P remobilization in relation to interplay between RNase and APase activities of vacuolar and cell wall compartments.

Up-regulation of intracellular and secreted APase and RNase by leaves, roots, and cell suspension cultures is widely recognized as a ubiquitous response that helps P-deprived plants recycle and scavenge their precious P resources (Abel and Glund, 1987; Tran *et al.*, 2010*b*; Plaxton and Tran, 2011). Conversely, few studies have considered the contribution of these APases to P remobilization during senescence. However, optimizing P remobilization from senescing tissues using selective breeding and/or biotechnological strategies will probably make a significant improvement to the PUE of many crops, thereby triggering an important reduction in the use of polluting and non-renewable P-containing fertilizers in agriculture (Veneklaas *et al.*, 2012). Considerable variations exist in the ability of different plants to remobilize P from senescing tissues. For example, leaves of a commercial soybean cultivar only remobilized ~50% of their total P during senescence, and similar values have been reported for several other crops (Crafts-Brandner, 1992; Veneklass *et al*., 2012). In contrast, the P remobilization efficiency of senescing harsh hakea (~85% in leaves and proteoid roots) (Fig. 1B) and *Arabidopsis* (~80% in leaves) (Himelblau and Amasino, 2001) is comparatively high. As summarized in the model presented in Fig. 5, the current study and a previous study (Robinson *et al.*, 2012*b*) have correlated this remarkable P remobilization ability with the pronounced up-regulation of cell wall and intracellular APases and RNases. It is notable that leaf senescence of two phylogenetically distinct species that both display excellent P remobilization efficiencies was paralleled by the pronounced induction of intracellular and cell wall-targeted APase and RNase activities. This indicates that this phenomenon may be a universal feature of the complex suite of biochemical adaptations evolved by native plant species that typically inhabit P-deficient soils. It has also been also demonstrated that the pair of prominent APase isoforms up-regulated during senescence of harsh hakea leaves or proteoid roots are probable PAPs (Fig. 3B). Likewise, senescing *Arabidopsis* leaves also induce cell wall-targeted and vacuolar APase activity, which is primarily due to the action of AtPAP26 (Fig. 4) (Robinson *et al.*, 2012*b*). This further implicates AtPAP26 as the predominant PAP isozyme involved in *Arabidopsis* PUE. Abolishing AtPAP26 expression in *atpap26* knock-out plants led to dramatic (>90%) reductions in cell wall-localized and vacuolar APase activities, resulting in markedly impaired P remobilization during leaf senescence (Fig. 4) (Robinson *et al.*, 2012*b*).

To the best of the authors’ knowledge, the apparent involvement of cell wall-localized PAPs and RNases in remobilizing P during senescence has not been previously suggested. The induction of cell wall-targeted APase and RNase during senescence of harsh hakea and *Arabidopsis* suggests that these hydrolases function to remobilize extracellular P from leaked P_o_. Classic studies by Bieleski’s group demonstrated that significant levels of cytoplasmic P_o_ can leak during P deficiency, and failure to recapture this P would seriously diminish a plant’s PUE (Bieleski and Johnson, 1972). Cell wall-targeted APases up-regulated by P-deficient plants were hypothesized to recycle P_i_ from this leaked P_o_ (Barrett-Lennard *et al*., 1993; Robinson *et al.*, 2012*a*). Senescence involves the regulated catabolism of macromolecules, including conversion of membrane lipids to sugars; the progressive disintegration of the plasma membrane would also permit significant levels of P_o_ (e.g. P-esters and RNA oligonucleotides) to escape from the cytoplasm (Fig. 5) (Lim *et al.*, 2007). It will therefore be of considerable interest to assess the extent to which P remobilization efficiency of senescing leaves (and thus overall PUE) might be improved in crop plants overexpressing senescence-inducible, cell wall-targeted PAP and nuclease isozymes such as AtPAP26 and AtRNS2, respectively.

Many other proteins undoubtedly contribute to plant P remobilization efficiency during senescence, particularly plasma membrane P_i_ transporters involved in P_i_ translocation (Buchanan-Wollaston *et al.*, 2003; Lim *et al.*, 2007), as well as proteins that mediate the strength of P sinks, particularly developing seeds. For example, unlike their vegetative tissues, seeds of many south-western Australian Proteaceae such as harsh hakea contain relatively high P concentrations (≥10mg g^–1^ dry matter) (Denton *et al.*, 2007; Groom and Lamont, 2010). The current study augments several recent discoveries concerning fascinating metabolic adaptations of harsh hakea for survival in a severely P-impoverished habitat (Lambers *et al.*, 2012; Shane *et al.*, 2013; Sulpice *et al.*, 2014). However, much is still to be learned from these ‘champions of low P tolerance’, especially if the aim is to apply some of the knowledge gained for agriculture in a world that features a rapidly escalating population coupled with diminishing availability of rock phosphate reserves.

## Supplementary data

Supplementary data are available at JXB online.

Figure S1. Differences in cell wall thickness and dry matter content of leaves and proteoid roots of harsh hakea.

Figure S2. Immunoblot and SDS–PAGE analysis of intracellular and cell wall proteins extracted from harsh hakea or *Arabidopsis* leaves.

Figure S3. Phosphatase activity of partially purified HpPAP1 and HpPAP2 as a function of assay pH.

Table S1. Purification of APase from 20g of senescing harsh hakea leaves.

Table S2. Effects of various substances on the activity of partially purified HpPAP1 and HpPAP2 from senescing leaves of harsh hakea.

Supplementary Data

## Abbreviations:

APase: acid phosphatase
P: phosphorus
PAP: purple acid phosphatase
PEP: phosphoenolpyruvate
P_o_: organic-P
PUE: P use efficiency
PVP: polyvinylpyrrolidone
PVPP: polyvinyl(polypyrrolidone).

## References

[CIT0001] Abel S Glund K. 1987. Ribonuclease in plant vacuoles: purification and molecular properties of the enzyme from cultured tomato cells. Planta 172, 71–78 24225789 10.1007/BF00403030

[CIT0002] Albenne C Canut H Jamet E. 2013. Plant cell wall proteomics: the leadership of *Arabidopsis thaliana* . Frontiers in Plant Science 4, 1–17 23641247 10.3389/fpls.2013.00111 PMC3640192

[CIT0003] Bariola PA MacIntosh GC Green PJ. 1999. Regulation of S-like ribonuclease levels in *Arabidopsis*. Antisense inhibition of RNS1 or AtRNS2 elevates anthocyanin accumulation. Plant Physiology 119, 331–342 9880376 10.1104/pp.119.1.331 PMC32237

[CIT0004] Barrett-Lenard EG Dracup M Greenway H. 1993. Role of extracellular phosphatases in the phosphorus-nutrition of clover. Journal of Experimental Botany 44, 1595–1600

[CIT0005] Bieleski RL Johnson PN. 1972. External location of phosphatase-activity in phosphorus-deficient *Spirodela oligorrhiza* . Australian Journal of Biological Science 25, 707–720

[CIT0006] Borderies G Jamet E Lafitte C Rossignol M Jauneau A Boudart G Monsarrat B Esquerre-Tugaye M-T Boudet A Pont-Lezica R. 2003. Proteomics of loosely bound cell wall proteins of *Arabidopsis thaliana* cell suspension cultures: a critical analysis. Electrophoresis 24, 3421–3432 14595688 10.1002/elps.200305608

[CIT0007] Bozzo GG Raghothama KG Plaxton WC. 2002. Purification and characterization of two secreted purple acid phosphatase isozymes from phosphate-starved tomato (*Lycoperscion esculentum*) cell cultures. European Journal of Biochemistry 269, 6278–6286 12473124 10.1046/j.1432-1033.2002.03347.x

[CIT0008] Buchanan-Wollaston V Earl S Harrison E Mathas E Navabpour S Page T Pink D. 2003. The molecular analysis of leaf senescence – a genomics approach. Plant Biotechnology Journal 1, 3–22 17147676 10.1046/j.1467-7652.2003.00004.x

[CIT0009] Craft-Brandner SJ. 1992. Phosphorus nutrition influence on leaf senescence in soybean. Plant Physiology 98, 1128–1132 16668736 10.1104/pp.98.3.1128 PMC1080317

[CIT0010] De Campos MCR Pearse SJ Oliveira RS Lambers H. 2013. Downregulation of net phosphorus-uptake capacity is inversely related to leaf phosphorus-resorption proficiency in four species from a phosphorus-impoverished environment. Annals of Botany 111, 445–454 23293017 10.1093/aob/mcs299 PMC3579450

[CIT0011] Delhaize E Randall PJ. 1995. Characterization of a phosphate-accumulator mutant of *Arabidopsis thaliana* . Plant Physiology 107, 207–213 12228355 10.1104/pp.107.1.207 PMC161187

[CIT0012] Denton MD Veneklaas EJ Freimoser FM Lambers H. 2007. *Banksia* species (Proteaceae) from severely phosphorus-impoverished soils exhibit extreme efficiency in the use and re-mobilization of phosphorus. Plant, Cell and Environment 30, 1557–1565 10.1111/j.1365-3040.2007.01733.x 17944818

[CIT0013] Fennoy SL Jayachandran S Bailey-Serres J. 1997. RNase activities are reduced concomitantly with conservation of total cellular RNA and ribosomes in O_2_-deprived seedling roots of maize. Plant Physiology 115, 1109–1117 12223861 10.1104/pp.115.3.1109 PMC158575

[CIT0014] Groom PK Lamont BB. 2010. Phosphorus accumulation in Proteaceae seeds: a synthesis. Plant and Soil 334, 61–72

[CIT0015] Hillwig MS Contento AL Meyer A Ebany A Bassham DC MacIntosh GC. 2011. RNS2, a conserved member of the RNase T2 family, is necessary for ribosomal RNA decay in plants. Proceedings of the National Academy of Sciences, USA 108, 1093–1098 10.1073/pnas.1009809108 PMC3024651 21199950

[CIT0016] Himelblau E Amasino RM. 2001. Nutrients remobilized from leaves of *Arabidopsis thaliana* during leaf senescence. Journal of Plant Physiology 158, 1317–1323

[CIT0017] Hurley BA Tran HT Marty NJ Park J Snedden WA Mullen RT Plaxton WC. 2010. The dual-targeted purple acid phosphatase isozyme AtPAP26 is essential for efficient acclimation of Arabidopsis to nutritional phosphate deprivation. Plant Physiology 153, 1112–1122 20348213 10.1104/pp.110.153270 PMC2899917

[CIT0018] Lambers H Cawthray GR Giavalisco P. 2012. Proteaceae from severely phosphorus-impoverished soils extensively replace phospholipids with galactolipids and sulfolipids during leaf development to achieve a high photosynthetic phosphorus-use-efficiency. New Phytologist 196, 1098–1108 22937909 10.1111/j.1469-8137.2012.04285.x

[CIT0019] Lambers H Finnegan PM Laliberté E Pearse SJ Ryan MH Shane MW Veneklaas EJ. 2011. Phosphorus nutrition of Proteaceae in severely phosphorus-impoverished soils: are there lessons to be learned for future crops? Plant Physiology 156, 1058–1066 21498583 10.1104/pp.111.174318 PMC3135942

[CIT0020] Lambers H Shane MW Laliberté E Swarts ND Teste FP Zemunik G. 2014. Plant mineral nutrition. In: Lambers H, ed. Plant life on the sandplains in Southwest Australia, a global biodiversity hotspot. Crawley: UWA Publishing, 101–127

[CIT0021] Lers A Khalchitski A Lomaniec E Burd S Green PJ. 1998. Senescence-induced RNases in tomato. Plant Molecular Biology 36, 439–449 9484484 10.1023/a:1005993024161

[CIT0022] Lim PO Kim HJ Nam HG. 2007. Leaf senescence. Annual Review of Plant Biology 58, 115–136 10.1146/annurev.arplant.57.032905.105316 17177638

[CIT0023] Motomizu S Wakimoto T Toei K. 1983. Spectrophotometric determination of phosphate in river waters with molybdate blue and malachite green. Analyst 108: 361–367 10.1016/0039-9140(84)80269-6 18963579

[CIT0024] Plaxton WC Tran HT. 2011. Metabolic adaptations of phosphate-starved plants. Plant Physiology 156, 1006–1015 21562330 10.1104/pp.111.175281 PMC3135920

[CIT0025] Raven JA. 2012. Protein turnover and plant RNA and phosphorus requirements in relation to nitrogen fixation. Plant Science 188–189, 25–35 10.1016/j.plantsci.2012.02.010 22525241

[CIT0026] Robinson WD Carson I Ying S Ellis K Plaxton WC. 2012a. Eliminating the purple acid phosphatase AtPAP26 in *Arabidopsis thaliana* delays leaf senescence and impairs phosphorus remobilization. New Phytologist 196, 1024–1029 23072540 10.1111/nph.12006

[CIT0027] Robinson WD Park J Tran HT Del Vecchio HA Ying S Zins JL Patel K McKnight TD Plaxton WC. 2012b. The secreted purple acid phosphatase isozymes AtPAP12 and AtPAP26 play a pivotal role in extracellular phosphate-scavenging by *Arabidopsis thaliana* . Journal of Experimental Botany 63, 6531–6542 23125358 10.1093/jxb/ers309 PMC3504502

[CIT0028] Shane MW Cramer MD Funayama-Noguchi S Cawthray GR Millar AH Day DA Lambers H. 2004. Developmental physiology of cluster-root carboxylate synthesis and exudation in harsh hakea. Expression of phospho*enol*pyruvate carboxylase and the alternative oxidase. Plant Physiology 135, 549–560 15122030 10.1104/pp.103.035659 PMC429412

[CIT0029] Shane MW De Vos M De Roock S Cawthray GR Lambers H. 2003. Effect of external phosphorus supply on internal phosphorus concentration and the initiation, growth and exudation of cluster roots in *Hakea prostrata* R.Br. Plant and Soil 248, 209–219

[CIT0030] Shane MW Fedosejevs ET Plaxton WC. 2013. Reciprocal control of anaplerotic phosphoenolpyruvate carboxylase by *in vivo* monoubiquitination and phosphorylation in developing proteoid roots of phosphate-deficient harsh hakea. Plant Physiology 161, 1634–1644 23407057 10.1104/pp.112.213496 PMC3613444

[CIT0031] Shane MW Lambers H. 2005. Cluster roots: a curiosity in context. Plant and Soil 274, 99–123

[CIT0032] Sulpice R Ishihara H Schlereth A. 2014. Low levels of ribosomal RNA account for the very high photosynthetic phosphorus-use efficiency of Proteaceae species. Plant, Cell and Environment 37, 1276–1298 10.1111/pce.12240 PMC4260170 24895754

[CIT0033] Thomas H. 2013. Senescence, aging and death of the whole plant. New Phytologist 197, 696–711 23176101 10.1111/nph.12047

[CIT0034] Tran HT Hurley BA Plaxton WC. 2010a. Feeding hungry plants: the role of purple acid phosphatases in phosphate nutrition. Plant Science 179, 14–27

[CIT0035] Tran HT Qian W Hurley BA She Y-M Wang D Plaxton WC. 2010b. Biochemical and molecular characterization of AtPAP12 and AtPAP26: the predominant purple acid phosphatase isozymes secreted by phosphate-starved *Arabidopsis thaliana* . Plant, Cell and Environment 33, 1789–1803 10.1111/j.1365-3040.2010.02184.x 20545876

[CIT0036] Veljanovski V Vanderbeld B Knowles VL Snedden WA Plaxton WC. 2006. Biochemical and molecular characterization of AtPAP26, a vacuolar purple acid phosphatase up-regulated in phosphate-deprived *Arabidopsis* suspension cells and seedlings. Plant Physiology 142, 1282–1293 16963519 10.1104/pp.106.087171 PMC1630754

[CIT0037] Veneklaas EJ Lambers H Bragg J. 2012. Opportunities for improving phosphorus-use efficiency in crop plants. New Phytologist 195, 306–320 22691045 10.1111/j.1469-8137.2012.04190.x

[CIT0038] Witkowski ETF Lamont BB Walton CS Radford S. 2001. Leaf demography, sclerophylly and ecophysiology of two banksias with contrasting leaf life spans. Australian Journal of Botany 40, 849–862

[CIT0039] Wang L Lu S Zhang Y Li Z Du X Liu D. 2014. Comparative genetic analysis of *Arabidopsis* purple acid phosphatases AtPAP10, AtPAP12, and AtPAP26 provides new insights into their roles in plant adaptation to phosphate deprivation. Journal of Integrative Plant Biology 56, 299–314 24528675 10.1111/jipb.12184

