# Supplementary material for: Senescence-inducible cell wall and intracellular purple acid phosphatases: implications for phosphorus remobilization in Hakea prostrata (Proteaceae) and Arabidopsis thaliana (Brassicaceae)
Source: J Exp Bot. 2014 Aug 28;65(20):6097–106. doi: 10.1093/jxb/eru348 (PMC4203141; doi:10.1093/jxb/eru348)
Supplement: Supplementary Data [file supp_65_20_6097__index.html]

Senescence-inducible cell wall and intracellular purple acid phosphatases: implications for phosphorus remobilization in Hakea prostrata (Proteaceae) and Arabidopsis thaliana (Brassicaceae) — Senescence-inducible cell wall and intracellular purple acid phosphatases: implications for phosphorus remobilization in Hakea prostrata (Proteaceae) and Arabidopsis thaliana (Brassicaceae) — Senescence-inducible cell wall and intracellular purple acid phosphatases: implications for phosphorus remobilization in Hakea prostrata (Proteaceae) and Arabidopsis thaliana (Brassicaceae) — Supplementary Data 

# Senescence-inducible cell wall and intracellular purple acid phosphatases: implications for phosphorus remobilization in *Hakea prostrata* (Proteaceae) and *Arabidopsis thaliana* (Brassicaceae)

## Supplementary Data

Data files

**Files in this Data Supplement:**

- Supplementary Data - Supplementary Data
