# Supplementary material for: Senescence-inducible cell wall and intracellular purple acid phosphatases: implications for phosphorus remobilization in Hakea prostrata (Proteaceae) and Arabidopsis thaliana (Brassicaceae)
Source: J Exp Bot. 2014 Aug 28;65(20):6097–106. doi: 10.1093/jxb/eru348 (PMC4203141; doi:10.1093/jxb/eru348)
Supplement: Supplementary Data [file supp_eru348_jexbot130674_file001.pdf]

Journal of Experimental Botany

Senescence-inducible cell-wall and intracellular purple acid phosphatases: implications for phosphorus remobilization in *Hakea prostrata* (Proteaceae) and *Arabidopsis thaliana* (Brassicaceae)

Michael W Shane, Kyla Stigter, Eric Fedosejevs and William C Plaxton

Supplementary Figure S1. Differences in cell-wall thickness and dry matter content of leaves and proteoid rootlets of harsh hakea. Representative cross-sections of mature leaf (A) and proteoid rootlet (B) Bars = 50 µm. (C) Proteoid root and leaf dry matter content as a proportion of fresh weight. Y = young (expanding) leaves; NS = non-senescent, fully mature leaves; S = fully senesced (dead) leaves. All values represent the mean (±SE) of triplicate determinations on n=4 biological replicates.

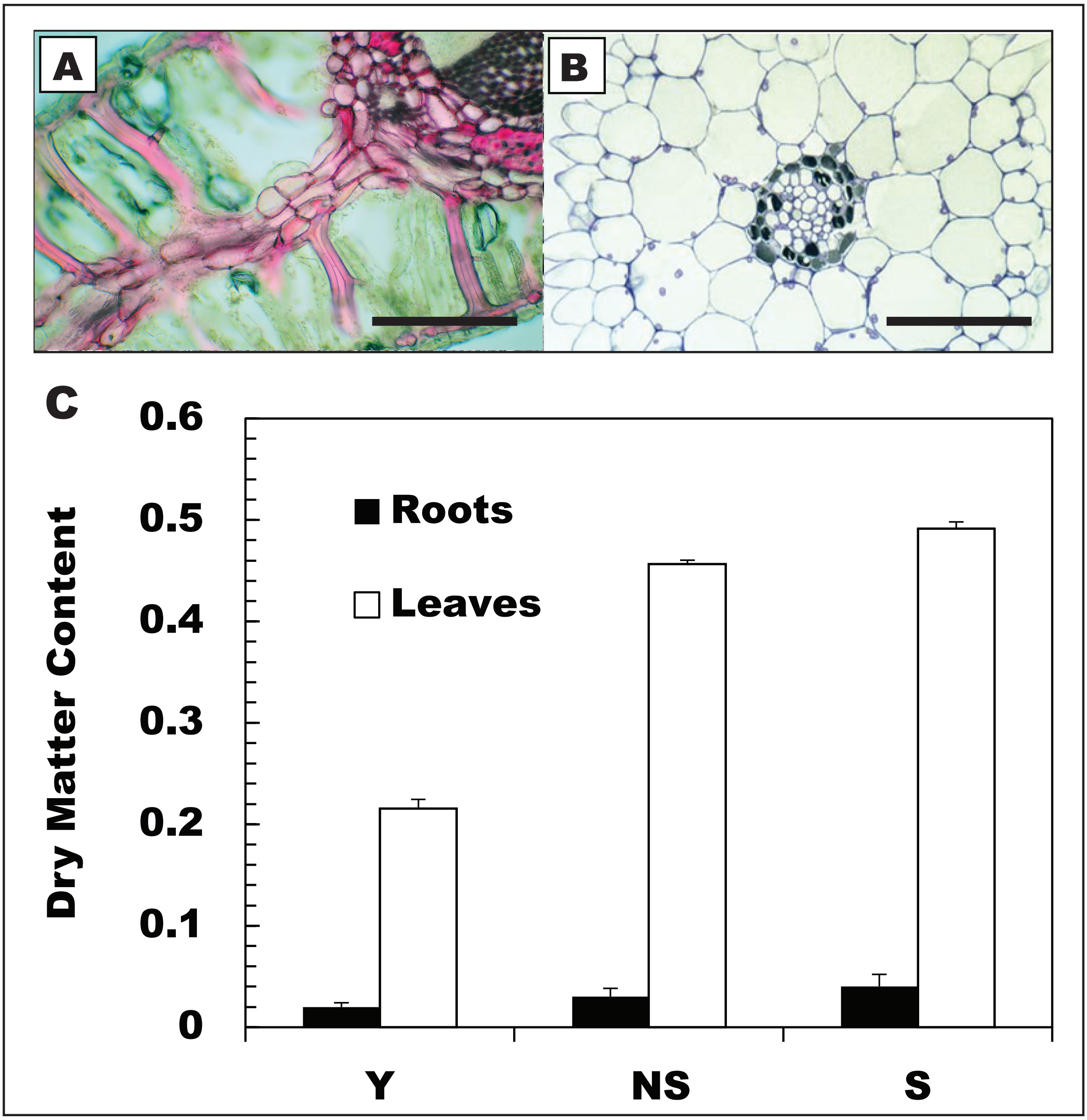

Supplementary Figure S2. Immunoblot and SDS-PAGE analysis of intracellular and cell-wall extracts isolated from harsh hakea or *Arabidopsis* leaves. Intracellular (IN) and cell-wall (CW) proteins extracted from harsh hakea (A, B) and *Arabidopsis* (C,D) leaves were resolved using 10% SDS-PAGE mini-gels (30 µg protein/lane) and subjected to immunoblot analysis using anti-(castor bean aldolase, Hodgson and Plaxton,1998) or anti-(castor bean PEP carboxylase, Uhrig *et al*., 2008) immune serum (A,C), or Sypro-Red staining (B,D) to visualize total protein. *M*, molecular mass standards. 'Pure PEPC' and 'Pure Ald' represent 100 ng of homogeneous PEP carboxylase (Uhrig *et al*., 2008) or aldolase (Hodgson and Plaxton,1998) isolated from endosperm of developing castor beans.

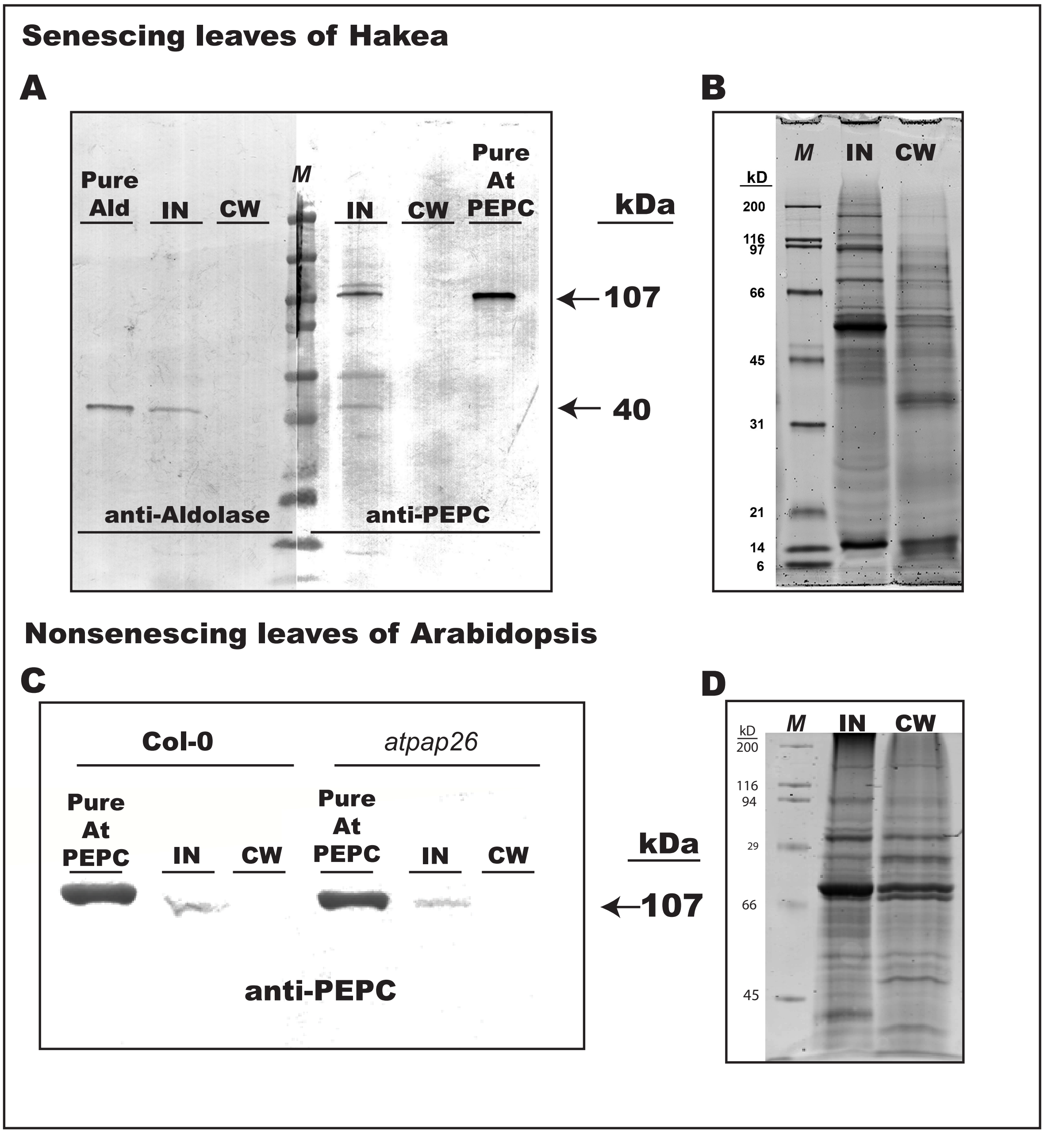

Supplementary Figure S3. Phosphatase activities of partially purified HpPAP1 and HpPAP2 as a function of assay pH. Assays were buffered by a mixture of 25mM sodium acetate, 25 mM MES, and 25 mM Bis-tris-Propane and employed 5 mM PEP as the substrate. All values represent the means (±SE) of *n*=4 separate determinations and were reproducible to within ±10% of the mean value.

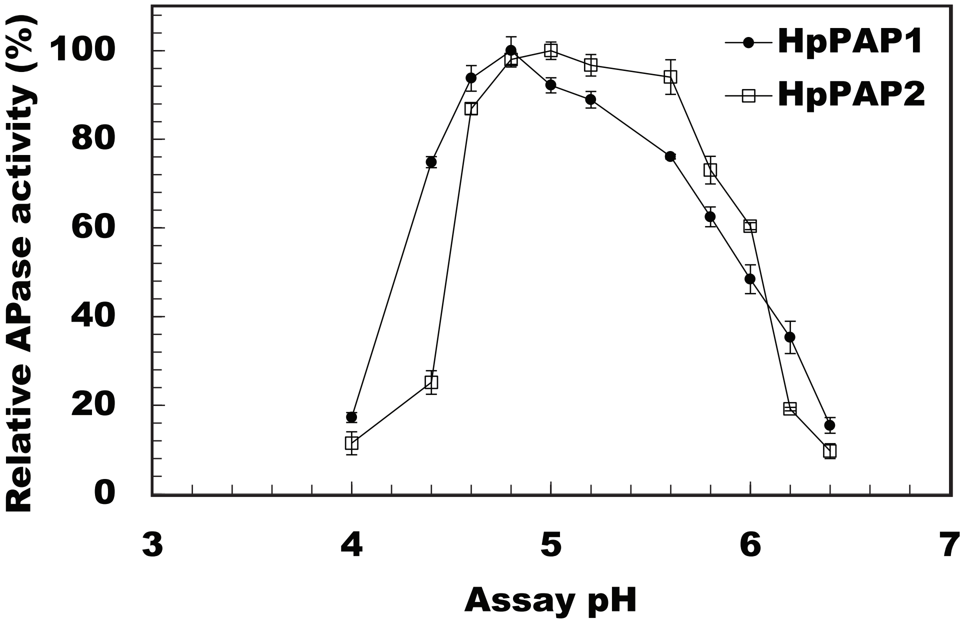

Supplementary Table S1. *Purification of APases from 20 g of senescing harsh hakea leaves*

Step	Total Volume	Activity	Protein	Specific Activity	Purification	Yield
	ml	*units*	*mg*	*Units mg^{-1}*	*-fold*	*%*
Clarified extract	55	13	258	0.05	1	100
$(NH_4)_2SO_4$ fractionation	21	5.4	63	0.08	1.6	42
Butyl-Sepharose*	3	0.87	4.8	0.18	3.6	7
Mono-S* (HpPAP1)	0.40	0.30	0.76	0.40	8	2
Mono-Q* (HpPAP2)	0.40	0.61	0.18	3.40	68	5

*concentrated fractions

Supplementary Table S2. *Effect of various substances on the activity of HpPAP1 and HpPAP2 partially purified from senescing leaves of harsh hakea*
The standard APase assay was used with 5 mM PEP as the substrate but lacking added $MgCl_2$. Enzyme activity in the presence of each substance (5 or 10 mM) is expressed relative to the respective control determined in the absence of any additions (except for 5 mM EDTA) and set at 100%. All values are means of *n*=3 independent determinations and are reproducible to within ±10% of the mean value.

	Relative Activity (%)			
	HpPAP1		***HpPAP2***	
**Addition**	5 mM	10 mM	5 mM	10 mM
$MgCl_2$	172	170	107	112
NaF	33	20	15	11
Vanadate	48	41	61	20
Molybdate	11	8	6	5
P_i	35	28	68	51
